# Neurotoxin-Induced Animal Models of Parkinson Disease: Pathogenic Mechanism and Assessment

**DOI:** 10.1177/1759091418777438

**Published:** 2018-05-29

**Authors:** Xian-Si Zeng, Wen-Shuo Geng, Jin-Jing Jia

**Affiliations:** 1 College of Life Sciences, Institute for Conservation and Utilization of Agro-Bioresources in Dabie Mountains, Xinyang Normal University, China

**Keywords:** Parkinson disease, neurotoxins, animal models, pathogenic mechanism, assessment

## Abstract

Parkinson disease (PD) is the second most common neurodegenerative movement disorder. Pharmacological animal models are invaluable tools to study the pathological mechanisms of PD. Currently, invertebrate and vertebrate animal models have been developed by using several main neurotoxins, such as 6-hydroxydopamine, 1-methyl-4-phenyl-1,2,3,6-tetrahydropyridine, paraquat, and rotenone. These models achieve to some extent to reproduce the key features of PD, including motor defects, progressive loss of dopaminergic neurons in substantia nigra pars compacta, and the formation of Lewy bodies. In this review, we will highlight the pathogenic mechanisms of those neurotoxins and summarize different neurotoxic animal models with the hope to help researchers choose among them accurately and to promote the development of modeling PD.

## Introduction

Parkinson disease (PD), affecting 1% of the population older than 65 years of age, is known as the second most common neurodegenerative disorder after Alzheimer’s disease. The disease is characterized by progressive loss of dopaminergic neurons in the substantia nigra pars compacta (SNc) of midbrain (Kikuchi et al., 2017) and the aggregation of α-synuclein and the formation of intraneuronal inclusions in remaining dopaminergic neurons, namely Lewy bodies which is a defining pathological characteristic of PD (Spillantini et al., 1997), accompanying with motor defects (static tremor, postural imbalance, bradykinesia, and muscle rigidity) and nonmotor symptoms(sleep disturbances, depression, and cognitive impairment; Chaudhuri et al., 2005; Langston, 2006). PD is clinically incurable since the pathological mechanism is not completely distinct.

The study of pathological mechanism is dependent on ideal animal models, which should reproduce all the clinical and pathological characteristics of PD. Epidemiological studies have revealed that familial forms account for few of PD subjects, while the overwhelming majority are sporadic forms (Schapira, 2008). Current animal models of PD can also be broadly divided into two categories: genetic and neurotoxic models, with the latter modeling sporadic PD (Blandini and Armentero, 2012). Various neurotoxin-based models of PD exhibiting notable degeneration of nigrostriatal dopaminergic neurons have been developed, such as 6-hydroxydopamine (6-OHDA), 1-methyl-4-phenyl-1,2,3,6-tetrahydropyridine (MPTP), paraquat, and rotenone. Unfortunately, none of those neurotoxic models perfectly reproduces all of the PD features. Putting aside the limitations, these pharmacological animal models have still contributed tremendously to our understanding of the disease processes and potential therapeutic targets in PD. Here, we will review these neurotoxin-induced models of PD, including invertebrate (*Drosophila*, *Caenorhabditis elegans*, and snail) and vertebrate (zebrafish, mouse, rat, and monkey) animals.

## Neurotoxins

It has been found that some structural analogs of dopamine, such as 6-OHDA and MPTP, could selectively lesion dopaminergic neurons and induce PD-like phenotypes. Epidemiological studies have confirmed that chronic exposure to agricultural chemicals (the herbicide paraquat and the pesticide rotenone) increase the risk of developing PD. These findings allow numerous groups to use the neurotoxins to generate PD models and to explore the mechanisms. The most common neurotoxins used to generate PD models are as following.

### 6-Hydroxydopamine

**Figure 1.**
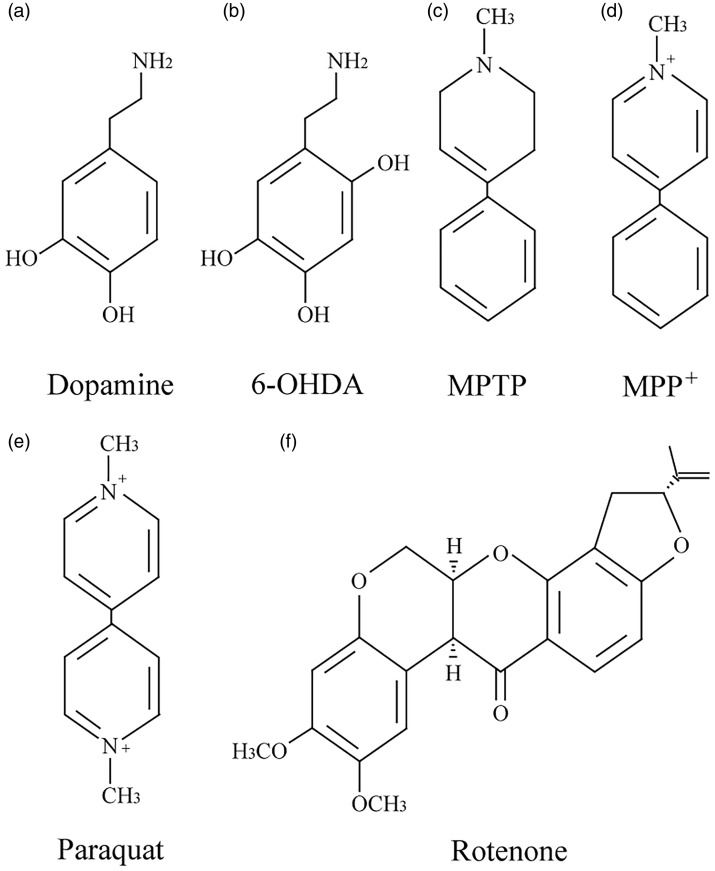
Structures of dopamine and main neurotoxins used to reproduce features of PD in animal models. 6-OHDA = 6-hydroxydopamine; MPTP = 1-methyl-4-phenyl-1,2,3,6-tetrahydropyridine; MPP^+^ = 1-methyl-4-phenylpyridinium ion.

**Figure 2.**
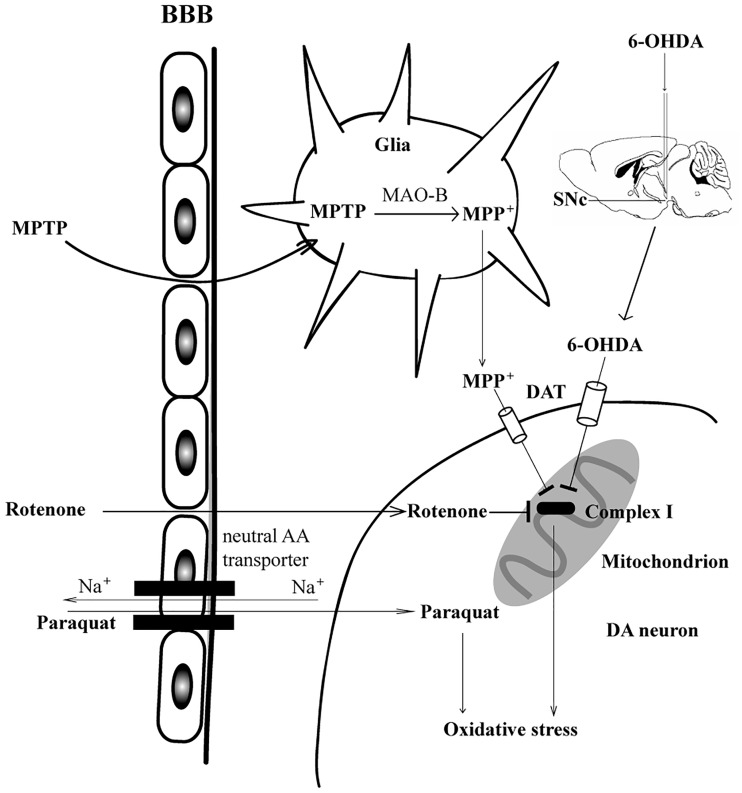
The administration routes and lesion mechanisms of dopaminergic neurotoxins. 6-OHDA must be delivered via intracerebral injection into SNc or striatum, where it is transported into dopaminergic neurons by DAT. MPTP, rotenone, and paraquat are systemically administrated because they can cross the BBB. MPTP is metabolized by MAO-B in glial cells to MPP^+^, which is also transported into dopaminergic neurons by DAT. Rotenone directly enters the dopaminergic neurons due to its hydrophobicity. 6-OHDA, MPP^+^, and rotenone inhibit the activity of complex I and further result in dopaminergic neurodegeneration. Neutral amino acid transporter mediates the Na^+^-dependent entry of paraquat into dopaminergic neurons, where it impairs the redox recycling and induces oxidative stress, ultimately leads to the neuronal death. 6-OHDA = 6-hydroxydopamine; MPTP = 1-methyl-4-phenyl-1,2,3,6-tetrahydropyridine; MPP^+^ = 1-methyl-4-phenylpyridinium ion.

The first animal model of PD is based on the intracerebral injection of 6-OHDA (Ungerstedt et al., 1974), which could induce mitochondrial dysfunction of dopaminergic neurons in the SNc. 6-OHDA cannot cross the blood–brain barrier (BBB), so it must be delivered by intracerebral injection. As an analog of dopamine (Figure 1(a) and (b)), 6-OHDA in SNc or striatum is transferred by dopamine transporter (DAT) into dopaminergic neurons and accumulates in mitochondria and then inhibits the activity of mitochondrial respiratory chain complex I (Figure 2; Glinka et al., 1996). Inhibition of monoamine oxidase A/B(MAO-A/B) enhanced the inhibitory of 6-OHDA on complex I, suggesting 6-OHDA itself, not its oxidation products, is responsible for the neurotoxicity (Glinka and Youdim, 1995). In addition, 6-OHDA can also inhibit the activity of mitochondrial complexes IV and decrease mitochondrial membrane potential (Glinka and Youdim, 1995; Prajapati et al., 2017). Once inside cells, 6-OHDA undergoes auto-oxidation or metabolic degradation and produces hydrogen peroxide, superoxide, and hydroxyl radicals. This process causes lipid peroxidation, protein oxidation, and DNA oxidation and finally results in oxidative stress and mitochondrial dysfunction (Hwang, 2013). 6-OHDA could trigger an increase in glutamate and δ-aminobutyric acid, and a decrease in glutamine in striatum, suggesting a likely shift in the steady-state equilibrium of the Gln-Glu cycle between astrocytes and neurons (Lei and Powers, 2013). The change in the Gln-Glu cycle would disrupt the balance between excitatory and inhibitory brain processes that would potentially lead to long-term abnormalities in glutamatergic and GABAergic activities.

### 1-Methyl-4-Phenyl-1,2,3,6-Tetrahydropyridine or 1-Methyl-4-Phenylpyridinium Ion

Langston et al. (1983) first discovered that MPTP induced striking parkinsonism in four drug abusers intravenously injected with an illicit drug containing the neurotoxin. MPTP could cross BBB and is metabolized by MAO-B in glial cells to the potent dopaminergic neurotoxin 1-methyl-4-phenylpyridinium ion (MPP^+^; Chiba et al., 1984; Schinelli et al., 1988), which is then transported into dopaminergic neurons by DAT because it is also a structural analog of dopamine (Figure 1(c) and (d)). Therefore, inhibition of MAO-B can block the neurotoxicity induced by MPTP (Johannessen et al., 1989; Adeyemo et al., 1993). Once transported into cells by the DAT, MPP^+^ is concentrated in the mitochondria, bind to NADH dehydrogenase, and inhibit the electronic transport from NADH to CoA (Ramsay et al., 1991). MPP^+^ selectively damages dopaminergic neurons due to its inhibiting effect on complex I, resulting in both reduction of ATP synthesis and accumulation of reactive oxygen species (ROS; Figure 2; Burns et al., 1985; Yan et al., 2013). MPTP could elevate Glu levels and key enzymes in the Gln-Glu cycle in the striatum, which are involved in neuron loss and motor function impairment (Lu et al., 2018). MPTP could replicate parkinsonism in a variety of species, including dogs, mice, primates, and humans (Burns et al., 1983; Burns et al., 1985; Johannessen et al., 1989; Zeng et al., 2014), so administration of MPTP is considered as a classic model of PD.

### Parquat

After MPTP/MPP^+^ was found to have the ability to produce parkinsonism, herbicide paraquat with similar structure to MPP^+^ has been used to model PD (Figure 1(e)). Paraquat can also cross the BBB. The pretreatment of L-valine, a high affinity substrate for the neutral amino acid transporter, markedly reduced the BBB penetration of paraquat, suggesting that the penetration process is mediated by the neutral amino acid transporter. This process is Na^+^-dependent and DAT-independent (Shimizu et al., 2001; McCormack and Di Monte, 2003). Paraquat has low affinity to mitochondrial complex I inside mitochondria, so paraquat does not inhibit complex I (Figure 2). Therefore, inhibition of complex I do not play a critical role in the neurotoxicity of paraquat. Paraquat *hijacks* the pentose phosphate pathway to increase NADPH reducing equivalents and stimulate paraquat redox cycling (Powers et al., 2017). As a redox cycling compound, paraquat induces oxidative stress by impairing the redox recycling of glutathione and thioredoxin (Ren et al., 2009; Niso-Santano et al., 2010), which inhibits the function of intracellular antioxidant systems. Paraquat also stimulates an increase in glucose uptake, the translocation of glucose transporters to the plasma membrane, and activation of AMP-activated protein kinase (Powers et al., 2017). The mitochondria-mediated apoptotic pathway is responsible for the pro-apoptotic activity of paraquat, leading to the upregulation of pro-apoptotic members of Bcl-2 family, followed by cytochrome c release and caspase-3 activation (Fei et al., 2008). Paraquat is highly selective for SNc dopaminergic neurons and leads to a 50% loss after multiple injections (Muthukumaran et al., 2014). Paraquat is only able to kill dopaminergic mesencephalic neurons in the presence of microglia (Peng et al., 2009). A single injection of paraquat is sufficient to activate microglia and predispose dopaminergic neurons to degenerate with the subsequent injections.

### Rotenone

Rotenone, a natural compound extracted from certain plant roots, is mainly used to kill vegetable and agricultural pests. Because it is highly hydrophobic (Figure 1(f)), rotenone could cross the BBB and biomembrane easily, which is not dependent on DAT. Once in the dopaminergic neurons, rotenone inhibits the activity of mitochondrial complex I, causing the production of ROS and mitochondrial dysfunction (Figure 2; Cannon and Greenamyre, 2013; Terron et al., 2018). Alterations to lipid and glutamine metabolism play an important compensatory role in response to complex I inhibition by rotenone(Worth et al., 2014). The rotenone model of PD has caught more attention since Betarbet et al. (2000) first developed a PD model based on continuous intravenous infusion of low-dose rotenone (3 mg/kg per day) for 33 days by subcutaneously implanting Alzet osmotic mini pumps at the back of Lewis rats, causing selective degeneration of dopaminergic neurons in nigrostriatum. However, another group has reported that intravenous infusion of rotenone (2.5 mg/kg/day for 28 days) in male Lewis rats showed multisystem degeneration, such as loss of striatal dopaminergic fibers (54%), nigral dopaminergic neurons (28.5%), striatal serotoninergic fibers (34%), striatal DARPP-32-positive projection neurons (26.5%), striatal cholinergic interneurons (22.1%), cholinergic neurons in the pedunculopontine tegmental nucleus (23.7%), and noradrenergic neurons in the locus ceruleus (26.4%; Hoglinger et al., 2003).

## Animal Models

Several invertebrate (*Drosophila*, *C. elegans*, and snail) and vertebrate (zebrafish, mouse, rat, and monkey) animals are used to model PD to research the pathogenesis as well as to discover new potential medicines. Many models successfully present the key features and clinical pathological characteristics of PD (Cannon et al., 2009).

### Drosophila

The short lifespan, simple maintenance, and the widespread availability of *Drosophila* allow scientists to research disease mechanisms and to develop potential drugs. Clusters of dopaminergic neurons are detectable in the larval and adult fruit fly. What’s more, the metabolic pathways of dopamine synthesis in *Drosophila* are similar to humans. Therefore, *Drosophila* has emerged as a suitable model for studying the mechanisms of neurodegeneration in PD. Treatment with paraquat induced PD-like symptoms in terms of oxidative stress, degeneration of dopaminergic neurons, locomotor defects, and apoptosis in flies (Shukla et al., 2014). After exposed to 500 μM rotenone-supplemented food for 3 days, the number of flies at the top section significantly decreased and the number of flies at the bottom section increased, suggesting that rotenone treatment markedly impaired the spontaneous locomotion activity (Liao et al., 2014; Basil et al., 2017). Interaction between genetic and environmental factors could aggravate the development of PD. The function of α-synuclein is thought to be related with the trafficking of synaptic vesicles and the regulation of vesicle exocytosis. α-synuclein mutants disrupt the clustering of synaptic vesicle mimics and result in neurodegeneration(Diao et al., 2013). Exposure to rotenone-supplemented food (low dose: 10 μM) of fly larvae expressing mutant α-synuclein A53T or A30P caused a much more severe PD symptoms (Varga et al., 2014; Jahromi et al., 2015), suggesting that the interaction between genetic and environmental factors contributes to the development of PD. The most acknowledged role of DJ-1 in the pathophysiology of PD is to protect neurons against oxidative stress (Biosa et al., 2017), as well as to maintain mitochondrial function (Thomas et al., 2011). Flies with PD-linked mutant of DJ-1 were also much more sensitive to the neurotoxicity of paraquat or rotenone and showed more severe defects in locomotor ability (Meulener et al., 2005; Park et al., 2005; Kumar et al., 2017).

### *C. elegans*

*C. elegans* is a 959-cell nematomorph with a simple nervous system which is composed of 302 neurons and a dopaminergic circuitry of exactly eight neurons, providing a framework for studying the mammalian brain (Varshney et al., 2011). *C. elegans* genes (*vps-35*, *lrk-1*, *pink-1*, *pdr-1*, and *djr-1*) express homologous to the accepted genetic risk factors linked to the familial form of human PD (respectively corresponding to the vesicular trafficking protein VPS35, the multidomain kinase LRRK2, the mitochondrial stress response proteins PINK1, Parkin, and DJ-1) and do not express α-synuclein (Nalls et al., 2014; Martinez et al., 2017). Therefore, *C. elegans* is highly suited to be used as a model animal to study PD. Overexpression of wild-type and mutated human α-synuclein (A53T, A30P) resulted in loss of dopaminergic neurons and accumulation of total and phosphorylated α-synuclein in cell bodies and dendrites (Lewy’s Bodies-like inclusions), as well as reduction in basal slowing response (Karpinar et al., 2009).

Substantial reports have showed that exposure of most neurotoxins, including 6-OHDA, parquat, rotenone, and MPP^+^, induced marked damage of mitochondrial DNA, decreased the mitochondrial DNA copies, and resulted in selective neurodegeneration of dopaminergic neurons in *C. elegans* (Nass et al., 2002; Zhou et al., 2013; Gonzalez-Hunt et al., 2014; Lehtonen et al., 2016; Offenburger et al., 2018). Either overexpression of α-synuclein or exposure to neurotoxins (6-OHDA, MPTP, rotenone, and paraquat) resulted in the loss of dopaminergic neurons for about 55% and 30%, respectively. Interaction between genetic and environmental exposures led to the death of dopaminergic neurons for 85% (Martinez et al., 2017), suggesting that gene-by-environment interaction enhanced the neurodegeneration of dopaminergic neurons in *C. elegans*.

### Snail

The pond snail, *Lymnaea stagnalis*, is widely used to study invertebrate neurobiology. The neurons in the central nervous system of snails are larger than Drosophila. Dopaminergic neurons have also been identified in the paired buccal ganglia of snails (Kabotyanski et al., 2000). Vehovszky et al. (2007) performed both acute test of rotenone (1–5 μM for 1–4 hr) and chronic test (0.5 μM for 4 days) on adult snails and found that rotenone induced significant reductions in spontaneous locomotion and feeding and decreased the tyrosine hydroxylase (TH) expression and dopamine level in the central nervous system. Maasz et al. (2017) selected young snail (not older than 3–4 months) and employed rotenone to induce PD model. The model snails were cultured in 0.5 µM rotenone solution (dissolved in dimethyl sulfoxide and added to the filtered natural lake water) for 4 days. They found that the locomotion, feeding activity, and dopamine level were all decreased in rotenone-treated snails compared with control snails. These studies demonstrate that snail is a suitable model for the study of PD based on analysis of the response of dopaminergic systems to rotenone at behavioral and cellular levels.

### Zebrafish

As a vertebrate animal, zebrafish is accordingly more closely related to humans than other invertebrate organisms, such as *Drosophila*, *C. elegans*, and snail. Zebrafish can be rapidly (3 months per generation) and prodigiously (up to one thousand offspring per female every week) bred. The dopaminergic neurons of adult zebrafish are well characterized in posterior tuberculum, which is homologous to the SNc in humans (Rink and Wullimann, 2001). Thus, zebrafish has become widely used to study the pathogenesis of PD. Adult zebrafish displayed behavioral alterations, including decreased locomotor activity in response to MPTP, whereas larval zebrafish exhibited developmental, behavioral, and dopaminergic sensitivity to MPTP, rotenone, or paraquat, suggesting that zebrafish could be a valuable model for investigating the molecular mechanisms underlying the neurotoxicity of PD-inducing agents (Bretaud et al., 2004). Intraperitoneal injection of MPTP (50 μg/5 μL, twice) showed reduction in number of crosses and swimming distance and increase in number of freezing bouts and freezing duration (Sarath Babu et al., 2016). Increasing evidence suggests that the neurotransmitter dopamine may have a neurotoxic metabolic product, selectively damaging dopaminergic neurons. L-DOPA’s therapeutic effect results from increasing brain dopamine concentrations. Dopamine is degraded into dopamine aldehyde (DOPAL) by MAO, and this may occur intracellularly or extracellularly after dopamine release. DOPAL is selectively toxic to neurons expressing DAT. Thus, L-DOPA has the potential to cause longer term damage to DAT-expressing neurons by augmenting the concentration of DOPAL or other oxidative products. L-DOPA treatment (1 mM) for 24 hr reduced the dopaminergic cell numbers in pretectum and ventral diencephalon, while neurons not expressing DAT were unaffected, and this was partially restored by inhibition of MAO. L-DOPA treatment also depressed the spontaneous swimming behavior of zebrafish at 5 days postfertilization (Stednitz et al., 2015). Zebrafish larvae immersed with 6-OHDA (250 μM) for 2 to 4 days postfertilization displayed a striking decrease in swimming distance and reduced expression of TH protein, *parkin*, and *pink1* mRNA (Feng et al., 2014).

Zebrafish is an ideally calculated model for drug screenings and investigating the pathological mechanisms underlying the death of dopaminergic neurons in PD. Loganin, an iridoid glycoside found in traditional Chinese medicines, such as *Flos lonicerae*, *Fruit cornus*, and *Strychonos nux vomica*, has been reported to regulate immune function and has anti-inflammatory and anti-shock effects. In a mice model, Loganin exerts neuroprotective effects on MPTP-treated mice by decreasing inflammation, autophagy, and apoptosis (Xu, Cui, et al., 2017). Loganin prevented both the loss of dopaminergic neurons and the defect of locomotor activity induced by MPTP in zebrafish larvae, possibly through the suppression of PI3K/Akt/mTOR axis and JNK signaling pathways (Yao et al., 2017). Treatment with rotenone (50 nM) for 6 hr in zebrafish at 48 hr postfertilization induced mitochondrial dysfunction and loss of TH-positive cells (Zhang, Nguyen, et al., 2017). Incubation with 6-OHDA (250 μM) for 2 to 4 days induced the loss of dopaminergic neurons and the deficiency of behavior movement in zebrafish at 1 to 3 days postfertilization, which could be reversed by some natural compounds, such as Danshensu and Berberine (Chong et al., 2013; Zhang, Li, et al., 2017). 11-dehydrosinulariolide, a marine-derived compound, is a cembranolide analog isolated from the soft coral *Sinularia flexibilis*. This compound restored the 6-OHDA-induced decrease of total swimming distance via upregulating DJ-1 expression and then activating the downstream Akt/PI3K, p-CREB, and Nrf2/HO-1 pathways in a zebrafish model of PD (Feng et al., 2016).

### Rodents

Being mammals and the low price, rodent models by neurotoxins are the most commonly used to study the pathogenesis of PD and to explore the mechanisms of neuroprotective compounds. In comparison, mouse model is more commonly used than rat model.

6-OHDA is more usually used to induce parkinsonism in rats than in mice because it is difficult to target small brain structures, such as the SNc or medial forebrain bundle (MFB). The coordination for stereotactic injection of 6-OHDA to the SNc of rats is 5.5 mm posterior, 2 mm left, and 8 mm ventral from bregma point (Maasz et al., 2017). Bilateral injection of 6-OHDA (low dose: 0.017 mg/kg) into SNc partially lesioned the nigral dopaminergic neurons and did not provoke motor deficits (Drui et al., 2014). 6-OHDA is frequently used as a unilateral model, because it has an attractive feature that each animal can serve as its own control as there is an unlesioned hemisphere. Unilateral injection of 6-OHDA (high dose: 0.032 mg/kg) into the SNc of rats significantly decreased the activity of mitochondrial complex I, contributing to the death of dopaminergic neurons directly via ROS mechanisms, including increased production of ROS and decreased ATP synthesis (Dabbeni-Sala et al., 2001; Ferrigno et al., 2015). The most common use of 6-OHDA is unilateral injection into the rat MFB. MFB lesion model is suitable to mimic PD and to research the specific functions of various striatal interneurons in the pathological process of PD. Unilateral injection of 6-OHDA in the right MFB of mice led to dopaminergic cell or fiber loss in the ipsilateral SNc and striatum (Park et al., 2018). Unilateral injection of 6-OHDA in MFB of rats also resulted in limb rigidity, cognitive, and mnemonic deficits, as well as a significant loss of TH-positive cells in the striatum or SNc (Ma et al., 2014). What’s more, the numbers of calretinin-positive and choline acetyltransferase-positive interneurons were notably reduced while these of neuropeptide Y-positive were markedly increased in the striatum, suggesting that dopamine depletion specifically regulated the vulnerability of striatal interneurons to the 6-OHDA-induced excitotoxicity in the MFB of rats. Some studies focus on the intrastriatal injection of 6-OHDA. After unilateral injection, the expression of TH and content of DA were significantly decreased in the striatum of rodents (Avagliano et al., 2016; Hegazy et al., 2017). Intrastriatal injection of 6-OHDA induced a retrograde degeneration of dopaminergic neurons within SNc and a decrease in TH-positive projection fiber density in the cingulate and motor cortex, suggesting that this model can be a promising tool to study the mechanisms of cortical pathology and cognitive decrease in PD (Becker et al., 2017). This route of administration displays the following advantages: (a) increasing the success rate in mice due to the large structure of the striatum; (b) inducing the progressive and less extensive lesion, which is more relevant to PD; and (c) producing nonmotor symptoms of PD, including cognitive, psychiatric, and gastrointestinal dysfunction. Endoplasmic reticulum stress and neuroinflammation were activated after 6-OHDA injection and contributed to the loss of dopaminergic neurons (Kumar et al., 2012; Hernandez-Baltazar et al., 2013; Avagliano et al., 2016; Table 1). Although it has been reported that 6-OHDA does interact with α-synuclein, 6-OHDA does not produce or induce proteinaceous aggregates or Lewy-like inclusions like those seen in PD (Blandini et al., 2008).

**Table 1.** The Administration Routes of Neurotoxins and the Parkinsonisms in Rodents.

Neurotoxins	Doses	Species	Routes	Pathology	Motor behavior	References
6-OHDA	0.032 mg/kg	Rat or mouse	i.c. into SNc	↓dopaminergic neurons in SNc,↓striatal DA	Impaired: postural asymmetry	(Dabbeni-Sala et al., 2001; Ferrigno et al., 2015)
	0.05 mg/kg	Rat	i.c. into MFB	↓TH-positive cells in the striatum or SNc	IMPAIRED: rigidity	(Park et al., 2018)
	0.02–0.16 mg/kg	Mouse	i.c. into MFB	↓TH-positive cells/fiber in the striatum or SNc	Impaired	(Ma et al., 2014)
	0.05–0.32 mg/kg	Rat or mouse	i.c. into striatum	↓striatal TH and DA,↓dopaminergic neurons within SNc, ↓TH fiber in motor cortex	Impaired	(Avagliano et al., 2016; Becker et al., 2017; Hegazy et al., 2017)
MPTP	33 nmol/24 hr	Rat	MPP^+^: i.c. into SNc/striatum	↓DA in SNc /↓dopaminergic neurons in SNc,↓striatal DA	Impaired: postural asymmetry and motor reduction	(McNaught et al., 1996)
	15–20 mg/kg	Mouse	i.p.	↓dopaminergic neurons in SNc, ↓striatal DA and TH fiber, no LB	Impaired: hypokinesia	(Zeng et al., 2014; Wu et al., 2015; Hwang et al., 2016; Xu, Langley, et al., 2017)
	46 mg/kg	Mouse	i.p. infusion	↓dopaminergic neurons in SNc, ↓striatal TH fiber, ↑LB	Impaired: hypokinesia	(Gibrat et al., 2009)
	23 mg/kg	Mouse	s.c.	no changes	Impaired: hypokinesia	(Fornai et al., 2005; Shimoji et al., 2005; Alvarez-Fischer et al., 2008)
Paraquat	10 mg/kg	Mouse	oral	↓TH-positive cells and mRNA level of DAT in SNc,↓striatal DA	Impaired	(Ren et al., 2009)
	10–15 mg/kg	Rat or mouse	i.p.	↓TH-positive cells and ↑α-synuclein in SNc,↓striatal dopaminergic nerve fibers, ↓striatal DA(contentious)	Impaired	(Manning-Bog et al., 2002; Muthukumaran et al., 2014)
Rotenone	2–3 mg/kg	Rat	s.c. infusion	↓dopaminergic neurons, ↑α-synuclein in SNc, peripheral toxicity	impaired: hypokinesia and rigidity	(Betarbet et al., 2000; Chen et al., 2015)
	5.0 μg	Rat	i.c.	↓dopaminergic neurons, ↑α-synuclein in SNc, no peripheral toxicity	Not determined	(Ravenstijn et al., 2008)
	2.75–3.0 mg/kg	Rat	i.p.	↓dopaminergic neurons, ↑aggregation of α-synuclein in SNc	Impaired: postural instability, rigidity, bradykinesia	(Cannon et al., 2009)
	50 mg/kg	Mouse	oral	↓TH-positive cells in SNc, ↑mitochondrial apoptosis	Not determined	(Chiu et al., 2015)

*Note.* DA = dopamine; DAT = dopamine transporter; i.c. = intracerebral; i.p. = intraperitoneal; LB = Lewy body; MFB = medial forebrain bundle; s.c. = subcutaneous; TH = tyrosine hydroxylase.

**Table 2.** MPTP-Induced Parkinsonism in Nonhuman Primates.

Species	Doses	Routes	Pathology	Motor behavior	References
Common marmosets	2 mg/kg	s.c.	Not determined	Impaired: reduction in basal locomotor activity, poor coordination of movement, abnormal and rigid posture, reduced alertness, and head-checking movements	(Lincoln et al., 2016)
Cynomolgus monkeys	0.4 mg/kg	i.v.	↓TH-positive dopaminergic neurons in SNc	Impaired: tremor, bradykinesia, and impaired balance	(Kikuchi et al., 2017)
Rhesus monkeys	0.2 mg/kg	not shown	↓tyrosine hydroxylase-positive neurons in SNc	Impaired: posture and resting tremor, rigidity, bradykinesia, and gesture instability	(Shi et al., 2017)
Squirrel monkeys	1.75 mg/kg	s.c.	↓TH-positive dopaminergic neurons in SNc, ↑α-synuclein	Impaired	(Purisai et al., 2005)

*Note*. i.v. = intravenously; s.c. = subcutaneous; TH = tyrosine hydroxylase.

MPTP can be used to model PD in rodents, particularly in mice. MPTP could induce oxidative stress, ROS, and energy failure, which are involved in the neuronal death. Increasing number of studies have suggested that mitochondrial dysfunction, activation of endoplasmic reticulum stress, and impaired autophagy are involved in MPTP-mediated neuronal apoptosis in SNc (Zeng et al., 2014; Giacoppo et al., 2017; Hu et al., 2017). MPTP mouse model is a classic model used to study the molecular pathways involved in PD neuronal cell death and to test the effectiveness of neuroprotectants. Rats are highly resistant to the neurotoxicity of MPTP because the high MAO activity in brain microvessels rapidly metabolize MPTP into polar MPP^+^, which has difficulty in traversing the BBB. However, rats are sensitive to MPP^+^ only when it is directly infused into the SNc or striatum (Galpern et al., 1996; Di Matteo et al., 2006). When injected into the striatum, MPP^+^ is accumulated in striatal dopaminergic nerve terminals and transported retrogradely to the SNc, resulting in the degeneration of dopaminergic neurons (Srivastava et al., 1993). MPP^+^(33 nmol/24 hr)-infused rats showed notable reduction in motor activity and displayed ipsilateral postural asymmetry, as well as significant decrease in the number of TH-positive cells in the ipsilateral SNc by about 90% (McNaught et al., 1996). The toxin is usually injected intraperitoneally to model PD through repeated administrations over a short period of time in mice, causing a selective lesion of dopaminergic neurons in SNc. Single intraperitoneal injection of MPTP only decreased the expression of TH without the loss of SNc dopaminergic neurons (Alam et al., 2017). Massive studies have reported that intraperitoneal injection for several days not only induced the death of dopaminergic neurons but also significantly impaired motor activity (Zeng et al., 2014; Wu et al., 2015; Hwang et al., 2016; Xu, Langley, et al., 2017). Unfortunately, this systemic intraperitoneal injection usually fails to produce the formation of Lewy body-like cytoplasmic inclusions. Interestingly, Gibrat et al. (2009) found that chronic intraperitoneal infusion of MPTP (46 mg/kg/day) for 14 days with osmotic mini-pumps reproduced the formation of neuronal inclusions as noted by the expression of α-synuclein within the cytoplasm of dopaminergic neurons in SNc, whereas the 28-day chronic intraperitoneal delivery (23 mg/kg/day), 7-day subacute intraperitoneal injection (20 mg/kg/day), and 28-day subcutaneous infusion (23 mg/kg/day) failed to induce the appearance of neuronal inclusions, which is accordant with previous studies (Fornai et al., 2005; Shimoji et al., 2005; Alvarez-Fischer et al., 2008; Table 1). The severity of nigrostriatal degeneration is as follows: 14-day intraperitoneal infusion (high dose) > 7-day intraperitoneal injection (low dose) > 28-day intraperitoneal infusion (low dose) > 28-day subcutaneous infusion (low dose). They considered that the low dose is possibly not sufficient to promote the formation of Lowy body. The formation of inclusions may be associated with the increased lactate levels in brain of mice treated with MPTP because it could activate AMP-activated protein kinase and facilitate α-synuclein accumulation and phosphorylation (Jiang et al., 2013). Currently, the MPTP mouse model is employed to test theories about cell death in PD, to study the neuronal death process, and to research other pathological effects of PD. It is also extremely useful as an initial screening tool to develop potential treatments for PD.

Paraquat is usually administrated orally or intraperitoneally. When taken orally for 4 months, paraquat (10 mg/kg) significantly impaired the locomotor activities, decreased the number of TH-positive cells and the mRNA level of DAT in SNc of mice, as well as reduced the level of dopamine in the striatum (Ren et al., 2009). This treatment markedly induced oxidative stress by increasing the content of malonaldehyde and by decreasing activities of antioxidant enzyme, such as superoxide dismutase and glutathione peroxidase. Oral exposure to paraquat triggered the expression of phosphorylated α-synuclein in the enteric nervous system of young mice, supporting the new opinion that PD may start in the gut (Naudet et al., 2017). Intraperitoneal injection of 10 mg/kg paraquat once a week for 3 consecutive weeks reduced the motor activity, resulted in loss of striatal dopaminergic nerve fibers without a substantial decrease of striatal dopamine, and increased the expression and accumulation of α-synuclein in the SNc of C57BL/6 mice (Manning-Bog et al., 2002). The ability of paraquat to induce increases in α-synuclein and to induce LB-like structures in dopaminergic neurons of the SNc emphasizes its importance to PD researchers. Paraquat plus maneb is usually used to model systemic PD. Combination of intraperitoneal 10 mg/kg paraquat and 30 mg/kg maneb (twice weekly for 7 weeks) significantly reduced the content of striatal dopamine by more than 20% in mice (Kachroo and Schwarzschild, 2014). However, Hosamani et al. (2016) reported that one intraperitoneal administration of paraquat (higher dose: 15 mg/kg) for 48 hr not only elicited significant oxidative stress in various brain regions but also decreased the dopamine levels in striatum of young mice. In a rat model, five intraperitoneal injections of 10 mg/kg paraquat, once every five days, reduced the number of TH-positive cells for about 50% (Muthukumaran et al., 2014; Table 1). Interestingly, Smeyne et al. (2016) reported that intraperitoneal administration of paraquat, either once (20 mg/kg) or twice (10 mg/kg) weekly for 3 weeks, had no effect on the number of TH-positive neurons as well as on microglial activation in the SNc.

Some rotenone models of rodents by different administration routes have been developed. The highlight of the use of chronic rotenone treatment of rats to model PD has been the generation of proteinaceous inclusions in some of the surviving dopaminergic neurons that cannot be found in the standard 6-OHDA and MPTP models. Chronic systemic administration using osmotic pumps has been the most common delivery regimen. Betarbet et al. (2000) modeled PD by subcutaneous infusion of rotenone (3 mg/kg per day) in rats and demonstrated that Lewis rats have less variability and more consistent lesions than Sprague Dawley rats. Chen et al. (2015) developed all the essential features of PD in Sprague Dawley rats subcutaneously infused with rotenone (2 mg/kg per day) for 5 weeks, including a strong increase in catalepsy score and a decrease in motor coordination activity, selective increase in oxidative damage in the striatal region, and massive degeneration of dopaminergic neurons in the SNc. The difference may be due to the longer infusion duration in the latter (5 weeks vs. 4 weeks). However, Ravenstijn et al. (2008) presented a negative report that subcutaneous infusion of rotenone (3 mg/kg per day) for up to 28 days failed to lesion dopaminergic neurons and only led to extensive toxicity in peripheral organs of Lewis rats (Ravenstijn et al., 2008). Interestingly, subcutaneous injection of rotenone (5–15 mg/kg) also had not been successful to lesion mice (Thiffault et al., 2000). Intracerebral infusion of rotenone (5.0 μg) produced a progressive death of dopaminergic neurons in SNc without associated peripheral toxicity and increased the expression and aggregation of α-synuclein (Ravenstijn et al., 2008). Male Lewis rats intraperitoneally injected with rotenone (2.75 and 3.0 mg/kg per day) for 60 days eventually exhibited severe PD-like phenotypes, including motor defects (bradykinesia, postural instability, or rigidity), nearly 50% loss of TH-positive neurons and aggregation of α-synuclein in SNc (Cannon et al., 2009). Oral administration of rotenone appears to cause little neurotoxicity, so the dose is usually high. Oral administration (50 mg/kg per day) for 14 days also induced the death of TH-positive cells in mouse SNc, the increase of ROS accumulation, mitochondrial membrane potential depolarization, and the activation of mitochondrial apoptotic pathway (Chiu et al., 2015; Table 1).

### Nonhuman Primates

Nonhuman primate models of PD have proven essential for understanding the molecular and cellular pathogenesis of the disease. MPTP is the most used neurotoxin to model PD in nonhuman primates. MPTP monkey model is mainly used to discern behavioral and symptomatic components of PD. Monkeys also represent the last level of PD treatment research prior to any treatment being administered to humans (Bezard and Przedborski, 2011). Nonhuman primates treated with MPTP develop motor defects that closely resemble the behaviors in PD, including bradykinesia, rigidity and postural abnormalities, as well as other pathophysiological changes observed in PD patients, so the MPTP-treated monkey has been recognized as the gold standard model of PD (Masilamoni and Smith, 2017). Common marmosets were subcutaneously administrated with MPTP (2.0 mg/kg) once daily for 5 days and exhibited significant and stable motor deficits, including a marked reduction in basal locomotor activity, poor coordination of movement, abnormal or rigid posture, reduced alertness, and head-checking movements (Lincoln et al., 2016). The adult male cynomolgus monkeys, intravenously injected with MPTP (0.4 mg/kg) twice a week for more than 12 weeks, displayed stable parkinsonian symptoms, such as tremor, bradykinesia, and impaired balance (Kikuchi et al., 2017). Rhesus monkeys were daily injection with a low dose of MPTP (0.2 mg/kg per day) and then accepted the neurotoxin by every 2 to 4 days. All the PD monkeys displayed mild PD symptoms after 9 weeks and reached a stable and classic parkinsonism stage after 18 weeks (Shi et al., 2017). In this primate model, TH-positive neurons in SNc were markedly decreased after MPTP injection. The authors also found that the mRNA expression of genes associated to PD, such as LRRK2, Parkin, and PINK1, was notably upregulated in SNc and peripheral blood, as well as DJ-1 expression was significantly decreased.

In addition to the profound motor symptoms and severe degeneration of dopaminergic neurons, the expression of α-synuclein was upregulated in SNc of squirrel monkeys after a single subcutaneous injection of 1.75 mg/kg MPTP (Purisai et al., 2005; Table 2). The toxic injury induced by MPTP in squirrel monkeys could promote the α-synuclein modifications, such as nitration and phosphorylation at Ser129, which are involved in the accumulation of insoluble α-synuclein and are implicated in the pathogenesis of PD (McCormack et al., 2008). It has been demonstrated that α-synuclein aggregates in dopaminergic neurons in the midbrain of chronic MPTP-treated young adult squirrel monkeys, suggesting that chronic MPTP treatment promotes the formation of α-synuclein-positive inclusions (McCormack et al., 2008). Another research group found that chronic and prolonged MPTP treatment (12–14 injections, 0.3 mg/kg, intravenously) induced the accumulation and phosphorylation of α-synuclein in the few remaining neuronal bodies in the SNc of cynomolgus monkeys but failed to found typical Lewy body (Halliday et al., 2009).

## Conclusions

PD has different phenotypes and potential etiologies, and it is accepted that PD is the result of the interaction between heredity and environment. Currently, various neurotoxic models of PD are available, in which substantial nigrostriatal degeneration is generally obtained, as well as PD motor symptoms are well replicated. Each model has advantages and disadvantages as we have discussed in this article. Research purpose will decide which model should be selected. In general, MPTP is the standard bearer for toxin-based PD animal models because the formation of α-synuclein-positive inclusions is also successfully induced in rodent and nonhuman primate models. How to build an ideal experimental model to reproduce all the phenotypic and pathological features of PD remains to be the key and difficult points. Such a model would be instrumental for a full understanding of PD pathogenesis. In my opinion, with the wide use of nonhuman primates and incessant innovation of administration routes of neurotoxin, a perfect model would be developed under the unremitting efforts of researchers. Based on this, PD will be conquered in the future.
